# Changing epidemiology of acute kidney injury in critically ill patients with COVID-19: a prospective cohort

**DOI:** 10.1186/s13613-022-01094-6

**Published:** 2022-12-28

**Authors:** Nuttha Lumlertgul, Eleanor Baker, Emma Pearson, Kathryn V. Dalrymple, Jacqueline Pan, Anup Jheeta, Kittisak Weerapolchai, Yanzhong Wang, Richard Leach, Nicholas A. Barrett, Marlies Ostermann

**Affiliations:** 1 Department of Critical Care, King’s College, Guy’s & St Thomas’ Hospital, NHS Foundation Trust, 249 Westminster Bridge Road, London, SE1 7EH UK; 2 Division of Nephrology and Excellence Centre for Critical Care Nephrology, King Chulalongkorn Memorial Hospital, Bangkok, Thailand; 3 Centre of Excellence in Critical Care Nephrology, Faculty of Medicine, Chulalongkorn University, Bangkok, Thailand; 4 Department of Population Health Sciences, King’s College London, London, UK; 5 Department of Urology, Guy’s and St Thomas’ NHS Foundation Trust, London, UK

**Keywords:** Acute kidney injury, COVID-19, Critically ill, Kidney replacement therapy, Wave

## Abstract

**Background:**

Acute kidney injury (AKI) is common in critically ill patients with coronavirus disease-19 (COVID-19). We aimed to explore the changes in AKI epidemiology between the first and the second COVID wave in the United Kingdom (UK).

**Methods:**

This was an observational study of critically ill adult patients with COVID-19 in an expanded tertiary care intensive care unit (ICU) in London, UK. Baseline characteristics, organ support, COVID-19 treatments, and patient and kidney outcomes up to 90 days after discharge from hospital were compared.

**Results:**

A total of 772 patients were included in the final analysis (68% male, mean age 56 ± 13.6). Compared with wave 1, patients in wave 2 were older, had higher body mass index and clinical frailty score, but lower baseline serum creatinine and C-reactive protein (CRP). The proportion of patients receiving invasive mechanical ventilation (MV) on ICU admission was lower in wave 2 (61% vs 80%; *p* < 0.001). AKI incidence within 14 days of ICU admission was 76% in wave 1 and 51% in wave 2 (*p* < 0.001); in wave 1, 32% received KRT compared with 13% in wave 2 (*p* < 0.001). Patients in wave 2 had significantly lower daily cumulative fluid balance (FB) than in wave 1. Fewer patients were dialysis dependent at 90 days in wave 2 (1% vs. 4%; *p* < 0.001).

**Conclusions:**

In critically ill adult patients admitted to ICU with COVID-19, the risk of AKI and receipt of KRT significantly declined in the second wave. The trend was associated with less MV, lower PEEP and lower cumulative FB.

*Trial registration*: NCT04445259.

**Supplementary Information:**

The online version contains supplementary material available at 10.1186/s13613-022-01094-6.

## Introduction

Since 2019, the world has faced multiple waves of severe acute respiratory syndrome coronavirus 2 (SARS-CoV-2) infections. Different viral strains, new diagnostics, therapies and vaccines have impacted the phenotype and course of the disease. Despite these advances, Coronavirus Disease 2019 (COVID-19) continues to pose significant challenges for patients, healthcare providers and healthcare systems [1–5].

Acute kidney injury (AKI) is a common complication of COVID-19, affecting 25–77% of patients admitted to an intensive care unit (ICU) and associated with major challenges for the healthcare team [4, 6–10]. Between 5 and 44% of patients receive kidney replacement therapy (KRT) [11–16]. We hypothesised that the incidence of COVID-19 associated AKI and KRT rates had declined since the beginning of the pandemic [17–21]. The aims of this study were to compare the epidemiology of COVID-19-associated AKI between the first and second wave of the pandemic and to identify key contributing factors.

## Materials and methods

### Setting, population, and ethical approval

This was a single-centre prospective analysis of critically ill COVID-19 patients admitted to a university-affiliated tertiary care hospital in London, between March 1st 2020 and February 28th 2021. The centre has 64 critical care beds, but capacity was expanded to 220 beds at the peak of the second wave.

We included adult (≥ 18 years) patients with COVID-19 confirmed by real-time reverse transcriptase polymerase chain reaction on nasopharyngeal or endobronchial samples. We excluded (1) patients with pre-existing end stage kidney disease (ESKD), (2) kidney transplant recipients, and (3) patients in whom COVID-19 was not the primary cause of ICU admission. In case of multiple admissions, only the first was included.

Ethical approval was obtained from the Health Research Authority and the Research Ethics Committee Health and Care Research Wales (REC Reference 20/WA/0175). Informed consent was obtained from the patients, personal, or professional consultee. The study was registered on clinicaltrials.gov (NCT04445259), conducted according to the Declaration of Helsinki and reported using the Strengthening the Reporting of Observational Studies in Epidemiology (STROBE) guidelines.

### Data collection

The details of data collection were previously published [11]. In brief, baseline characteristics, comorbidities, laboratory parameters and organ support on admission were collected. COVID-19 treatments (e.g. immunomodulatory therapies, antivirals, proning, anticoagulation, etc.), daily fluid intake and output (day 1–7), daily serum creatinine (SCr) and urine output until day 14 and complications during hospital admission were recorded. Data were obtained through manual chart reviews by trained researchers and subsequently verified. Laboratory parameters were extracted from electronic health records. SCr results after hospital discharge were obtained from local care records or by contacting general practitioners. The last follow-up date was August 31st 2021.

Patients were grouped into wave 1 or 2. Wave 1 included the period March–August 2020, and wave 2 referred to September 2020–February 2021 [21]. The Kidney Diseases: Improving Global Outcomes (KDIGO) classification was used to define and stage AKI using both SCr and urine output criteria [22]. In obese patients, we calculated hourly urine output based on ideal body weight [23]. Baseline SCr was determined from the lowest outpatient SCr values between 7 and 365 days before ICU admission [24]. If a historical SCr result was unavailable, we used the lower values between the first SCr on hospital admission or SCr derived from the back-calculation of an estimated glomerular filtration rate (eGFR) of 75 mL/min/1.73 m^2^ using the Modification of Diet in Renal Disease (MDRD) formula [22]. New-onset AKI was defined as AKI which developed more than 48 h after ICU admission.

Kidney recovery was defined as having SCr < 1.5 times baseline value and being dialysis independent and alive [25]. Patients with AKI were divided into 3 groups according to AKI duration from the day of AKI diagnosis until kidney recovery; transient (≤ 2 days), persistent-medium (3–6 days), and persistent-long duration (≥ 7 days or non-recovery) [26]. Cumulative fluid balance (FB) (%) was calculated as [total fluid intake (L) – total output of all body fluids (L) × 100] divided by body weight on admission (kg) [27]. Chronic kidney disease (CKD) was defined as eGFR < 60 mL/min/1.73 m^2^ [28].

### Outcomes

The primary outcome was the occurrence of AKI within 14 days after ICU admission. Secondary outcomes were the differences between wave 1 and 2 of the following: (1) KRT rate within 14 days after ICU admission; (2) risk factors for overall AKI and KRT; (3) risk factors for new AKI and KRT after 48 h, and (4) patient and kidney outcomes at ICU and hospital discharge, and at 90 days.

### Statistical analysis

Baseline characteristics, complications, COVID-19 treatments, kidney outcomes and mortality were stratified by wave. Binary and categorical variables are presented using counts and percentages. The distributions of continuous variables were assessed using coefficients of skewness and summarised as either mean and standard deviation (SD) for normally distributed variables or median and interquartile range (IQR) for non-normally distributed variables. To assess for differences between the waves, binary or categorical variables were assessed using the Chi-square test. Mann–Whitney *U* test or *t *test were undertaken for continuous data depending on the distribution.

AKI incidence rates within 14 days after ICU admission are presented as cumulative incidence and events/100 person-days, using mortality and ICU discharge before 14 days as competing risks. Cox proportional hazards models were used to estimate hazard ratios (HRs) with 95% confidence interval (CI) for 90-day mortality with AKI modelled as a time-varying covariate.

Univariate and multivariate logistic regression were used to examine the relationship between demographics (exposures) and AKI or KRT (outcomes). The multivariate models were all adjusted for new systemic steroid therapy, remdesivir, interleukin (IL)-6 antagonists and invasive vs non-invasive ventilation. Further adjustments were made for COVID-19 wave (1 vs. 2), age, gender, and ethnicity where indicated. Regression coefficients are represented as odds ratios (OR) with a 95% CI.

To explore the relationship between cumulative FB at 48 h after ICU admission and the development of new AKI and KRT, we differentiated between 5 FB groups (< − 2%, − 2% to 0%, 0% to + 2% (reference group), + 2% to + 4% and >  + 4%). Groups were combined if the number of patients in one category was small. Patients with AKI or KRT within 48 h of admission were excluded from the model investigating new AKI and KRT, respectively.

### Sensitivity analysis

To further explore the association between 24-h cumulative FB and AKI and KRT, we performed a sensitivity analysis by excluding patients with AKI or KRT within 24 h of hospital admission.

## Results

### Baseline characteristics, ICU management and complications

**Fig. 1:**
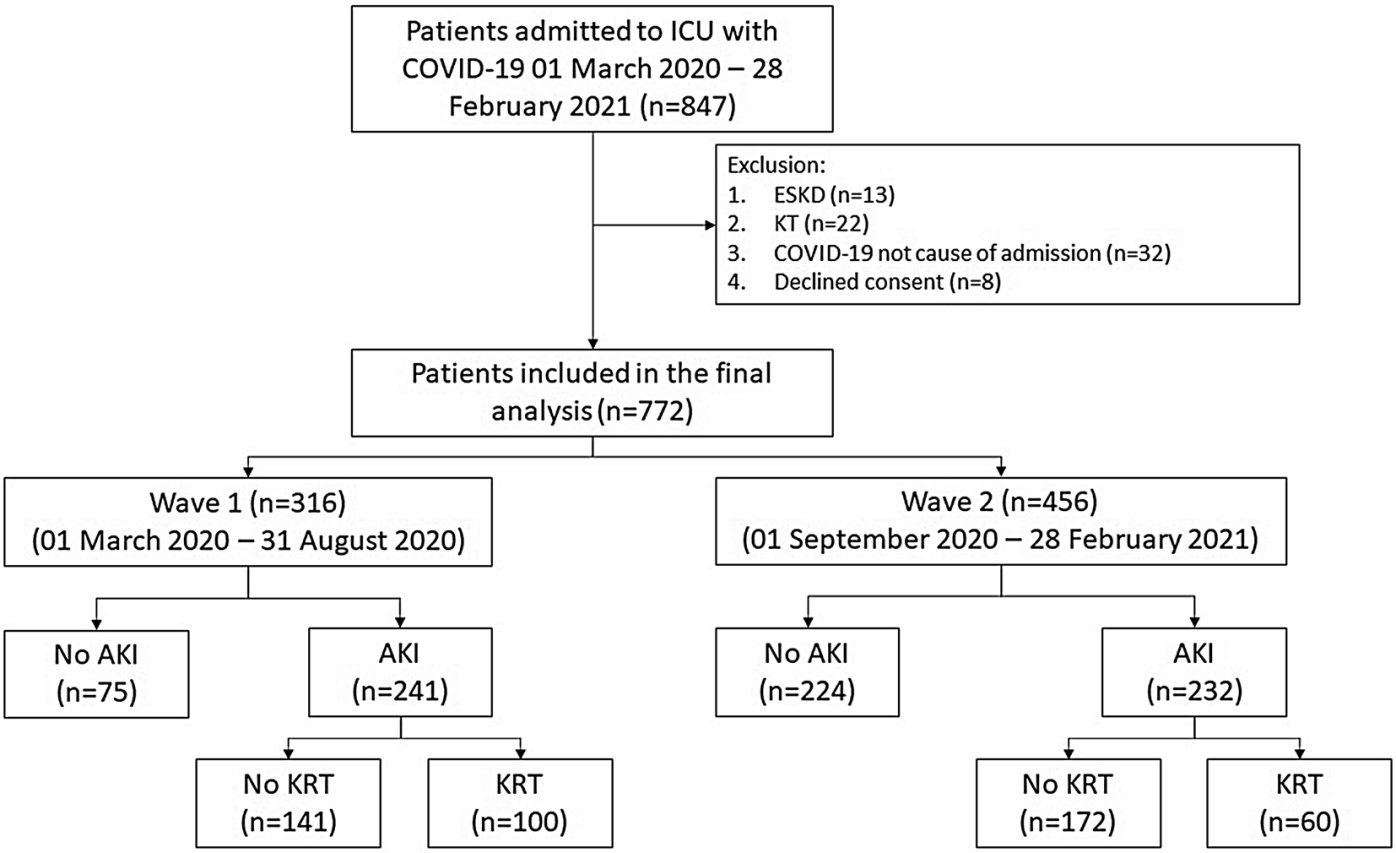
Study flowchart. *ICU* intensive care unit, *ESKD* end-stage kidney disease, *KT* kidney transplantation, *AKI* acute kidney injury, *KRT* kidney replacement therapy

Between March 1st 2020 and February 28th 2021, 847 critically ill SARS-CoV-2 positive patients were admitted to ICU. A total of 772 patients were included in the final analysis: 316 (40.9%) from the first wave and 456 (59.1%) from the second wave (Fig. 1). Compared with wave 1, patients admitted to ICU in wave 2 were older, had a higher body mass index (BMI), lower Sequential Organ Failure Assessment (SOFA) score, higher clinical frailty score and were less likely to be of black ethnicity (Table 1). In wave 2, patients had higher neutrophil counts and lower baseline SCr, ferritin, C-reactive protein (CRP), PaO_2_/FiO_2_ ratio and lymphocyte counts at ICU admission. They were less likely to receive invasive MV (61% vs. 80%; *p* < 0.001) and vasopressor support (30% vs. 42%; *p* < 0.01) on admission compared with patients in wave 1. In patients who received invasive MV, the maximum levels of positive end-expiratory pressure (PEEP) in the first 48 h were significantly lower in wave 2 than wave 1.

**Table 1:** Baseline characteristics, stratified by wave

	COVID wave 1 (*n* = 316)	COVID wave 2 (*n* = 456)
	Mean (SD)/median (IQR) or *N* (%)	
Age (years)	54.6 (14.0)	57.3 (13.2)***
Male sex	222 (70%)	297 (65%)
Ethnicity		
White	120 (38%)	179 (39%)***
Black	90 (29%)	72 (16%)
Other	106 (34%)	205 (45%)
Source of admission		
ED	94 (30%)	148 (32%)
Ward	105 (33%)	116 (25%)
Transfer from another hospital	114 (36%)	188 (41%)
Other	3 (1%)	4 (1%)
Infection setting		
Community	278 (88%)	425 (93%)**
Hospital	8 (2%)	18 (4%)
Occupational	24 (8%)	10 (2%)
Other	6 (2%)	3 (1%)
BMI	28.4 (24.8–33.6)	29.4 (26.0–36.2)**
Current smoker	13 (4%)	17 (4%)
Days of symptoms pre-admission	9 (7–13)	9 (5–13)
Admission SOFA score	5 (3–7)	4 (3–6)***
APACHE II score	14.1 (4.8)	13.2 (5.1)
Clinical frailty score	2 (2–3)	3 (2–3)*
Pre-existing comorbidities		
Diabetes	96 (30%)	137 (30%)
Asthma	48 (15%)	69 (15%)
Hypertension	127 (40%)	205 (45%)
CAD	16 (5%)	41 (9%)*
CHF	14 (4%)	17 (4%)
Atrial fibrillation/atrial flutter	11 (3%)	12 (3%)
COPD	12 (4%)	35 (8%)
Chronic kidney disease	22 (7%)	33 (7%)
Chronic liver disease	12 (4%)	17 (4%)
HIV infection	7 (2%)	9 (2%)
Malignancy	14 (4%)	23 (5%)
Baseline laboratory parameters and organ support on admission to ICU		
Type of ventilation on admission		
Invasive ventilation	254 (80%)	278 (61%)***
Non-invasive ventilation	4 (1%)	16 (4%)
High-flow nasal cannula	24 (8%)	151 (33%)
None	34 (11%)	11(2%)
Extracorporeal membrane oxygenation	58 (18%)	64 (14%)
Number of vasopressors		
0	183 (58%)	320 (70%)**
1	131 (38%)	130 (29%)
2	11 (3%)	6 (1%)
3	1 (1%)	–
Baseline creatinine (µmol/L)	81.5 (68.0–96.1)	78.0 (65.5–91.5)*
pH	7.4 (7.3–7.4)	7.4 (7.4–7.5)***
PaO_2_ (kPa)	10.1 (8.8–12.8)	8.3 (7.2–9.6)***
Ionised calcium (mmol/L)	1.1 (1.1–1.2)	1.1 (1.1–1.2)
Lactate (mmol/L)	1.7 (1.3–2.3)	1.8 (1.4–2.4)
Chloride (mEg/L)	99.7 (5.5)	102.2 (5.3)***
White blood cells (10^9^/L)	8.7 (6.4 -12.4)	9.7 (6.5–13.8)
Neutrophils (10^9^/L)	7.5 (4.8–10.4)	8.4 (5.4–12.1)*
Lymphocytes (10^9^/L)	0.8 (0.5–1.1)	0.7 (0.4–1.0)**
Haemoglobin (g/L)	117 (21.3)	118 (23.9)
Ferritin (µg/L)	1121 (676–2182)	1039 (527–1859)*
d-dimer (mg/L)	1.7 (0.80–6.84)	2.1 (0.92–7.31)
CRP (mg/L)	170 (92–292)	91 (42–174)***
PaO_2_/FiO_2_ ratio	17.8 (13.1–25.1)	13 (10.0–17.7)***
On admission to ICU		
AKI diagnosis	121 (38%)	110 (24%)
Kidney replacement therapy	31 (13%)	20 (9%)**
During ICU admission		
Max PEEP in first 24 h (cmH_2_O)	7.5 (4.9)	5.5 (5.1)***
Max PEEP in first 48 h (cmH_2_O)	8.42 (4.84)	6.45 (5.26)***
AKI diagnosis	241 (76%)	232 (51%)***
Kidney replacement therapy	100 (32%)	60 (13%)***
Delta fluid balance between day 1 and 2 (mL/kg)^a^	3.64 (16.4)	4.01 (16.1)
Diuretic use in first 48 h (%)	111 (35%)	96 (21%)***

Binary and categorical variables are presented using counts and percentages. The distribution of continuous variables was assessed using coefficients of skewness and then summarised by mean and standard deviation (SD) or median and interquartile range (IQR) where appropriate. To assess for differences between the waves of data collection, binary or categorical variables were assessed using the chi-square test. Mann–Whitney *U* tests or *t *test were undertaken for continuous data depending on the distribution

*SD* standard deviation, *IQR* interquartile range, *N* number, *ED* emergency department, *BMI* body mass index, *SOFA* Sequential Organ Failure Assessment, *APACHE II* Acute Physiologic and Chronic Health Evaluation II, *CAD* coronary artery disease, *CHF* congestive heart failure, *COPD* chronic obstructive pulmonary disease, *HIV* human immunodeficiency virus, *CRP* C-reactive protein, *AKI* acute kidney injury, *ICU* intensive care unit, *PEEP* positive end-expiratory pressure

* *p* < 0.05

** *p* < 0.01

*** *p* < 0.001

^a^Calculated as net fluid balance on day 2 minus net fluid balance on day 1, divided by baseline body weight

During the first 14 days in ICU, patients in wave 2 were more likely to receive steroids (99% vs. 59%; *p* < 0.001), remdesivir (41% vs. 6%; *p* < 0.001) and IL-6 antagonists (27% vs. 1%; *p* < 0.001) than patients in wave 1 (Table 2). In those who received steroids, the median dose of dexamethasone or equivalent was higher in wave 2 than wave 1 (22.5 mg [IQR 13.2–39.6] vs. 18.8 mg [IQR 11.1–30]; *p* < 0.001). The use of therapeutic anticoagulation and proning were comparable. Overall complications were similar, except for lower incidence of acute respiratory distress syndrome and myopericarditis in wave 2.

**Table 2:** Complications, treatments, AKI and ICU outcomes, stratified by wave

	COVID wave 1 (*n* = 316)	COVID wave 2 (*n* = 456)
	Mean (range)/mean (SD)/*N*(%) or median (IQR)	
Treatment		
Remdesivir	19 (6%)	189 (41%)***
New systemic steroids	186 (59%)	450 (99%)***
Median dexamethasone or equivalent dose	18.8 (11.1–30.0)	22.5 (13.2–39.6)***
IL-6 antagonists	3 (1%)	122 (27%)***
Anakinra	25 (8%)	16 (4%)**
Neuromuscular blockade	150 (47%)	184 (40%)
Inhaled epoprostenol	54 (17%)	53 (12%)*
Inhaled nitric oxide	28 (9%)	49 (11%)
Proning position	121 (38%)	190 (42%)
Therapeutic anticoagulation	138 (44%)	194 (43%)
Complications		
Acute respiratory distress syndrome	235 (74%)	296 (65%)**
Congestive heart failure	16 (5%)	38 (8%)
Myopericarditis	19 (6%)	9 (2%)**
New infection^a^	176 (56%)	285 (63%)
Thrombosis	94 (30%)	141 (31%)
AKI		
Overall incidence	241 (76%)	232 (51%)***
Maximum staging: 1	64 (27%)	122 (53%)
2	41 (17%)	40 (17%)
3	136 (56%)	70 (30%)
AKI category		
Transient duration (≤ 2 days)	85 (35%)	151 (65%)***
Persistent-medium duration (3–6 days)	40 (17%)	23 (10%)
Persistent-long duration (≥ 7 days)	116 (48%)	57 (25%)
Kidney replacement therapy	100 (32%)	60 (13%)***
Patient outcomes		
Receipt of mechanical ventilation during ICU stay	269 (85%)	318 (70%)***
ICU mortality	89 (28%)	105 (23%)
Hospital mortality	92 (29%)	113 (25%)
ICU length of stay	13.5 (6–29)	13 (6–28)
Hospital length of stay	20 (11–42)	21 (12–45)
Kidney outcomes		
Dialysis dependence at 30 days	52 (17%)	39 (9%)***
Dialysis dependence at hospital discharge^b^	8 (4%)	7 (2%)
Serum creatinine at hospital discharge^b^ [µmol/L]	65 (52.5–88.5)	66 (52–83)
Kidney recovery at hospital discharge in AKI patients^b^	132 (83%)	136 (89%)**
Outcomes at 90 days after discharge		
Dialysis dependence^b^^,c^	9 (4%)	2 (1%)***
Serum creatinine^b^ (µmol/L)	73 (57–94)	70 (56–86)
CKD^b^	27 (14%)	23 (11%)
Mortality	93 (29%)	120 (26%)

Binary and categorical variables are presented using counts and percentages. The distribution of continuous variables was assessed using coefficients of skewness and then summarised by mean and standard deviation (SD) or median and interquartile range (IQR) where appropriate. Net fluid balance is presented as mean (range). To assess for differences between the waves of data collection, binary or categorical variables were assessed using the chi-square test. Mann–Whitney *U* test or *t *test were undertaken for continuous data depending on the distribution

*SD* standard deviation, *IQR* interquartile range, *N* number, *AKI* acute kidney injury, *ICU* intensive care unit, *CKD* chronic kidney disease, *IL-6* interleukin-6

^a^New infection was defined as a suspected or confirmed new bacterial infection other than COVID-19 that developed after admission to the ICU

^b^In survivors

^c^Not available in 25 patients in wave 1 and 7 patients in wave 2

^* ^p < 0.05

^**^p < 0.01

^***^p < 0.001

### AKI diagnosis, incidence, and characteristics

**Fig. 2:**
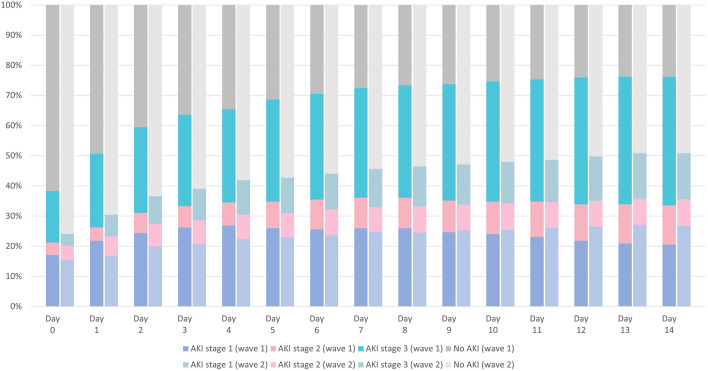
AKI staging by day after ICU admission and stratified by COVID-19 wave

True baseline SCr results were available for 278 patients (36%). AKI was defined by SCr in 27.7%, urine output in 20.9% and both criteria in 51.4%. On ICU admission, AKI was prevalent in 121 (38%) in wave 1 and 110 (24%) in wave 2 (*p* < 0.001). The overall AKI incidence within 14 days after ICU admission was 76% in wave 1 and 51% in wave 2 (*p* < 0.001) (Fig. 2). The cumulative incidence rate of AKI was 28.5 events/100-person days (95% CI 23.8–34.2) in wave 1 and 22.7 events/100-person days (95% CI 18.9–27.1) in wave 2, accounting for mortality and ICU discharge as competing risks. The numbers and proportions of AKI and KRT relative to ICU admission are shown in Additional file 1: Figs. S1, S2. The median day of AKI onset was 1 (IQR 0–5) in wave 1 and 4 (IQR 1–10) in wave 2. Patients admitted in wave 1 were more likely to have more severe and more prolonged AKI (Table 2). Characteristics between patients with and without AKI stratified by wave are shown in Additional file 1: Table S1.

Multivariate analysis demonstrates that age, BMI, admission SOFA score, invasive MV, higher baseline SCr, high CRP, low pH and low ionised calcium on admission were associated with AKI (Table 3; Additional file 1: Table S2).

**Table 3:** Adjusted associations between demographic characteristics and diagnosis of acute kidney injury for all patients and stratified by wave

	All participants^a^ (*n* = 772)	COVID wave 1 (*n* = 316)	COVID wave 2 (*n* = 456)
	Odds ratios (95% CI)		
Age^b^	1.02 (1.01, 1.03)***	1.02 (1.00, 1.05)**	1.02 (1.01, 1.04)***
Male sex^c^	1.07 (0.77, 1.51)	0.84 (0.46, 1.55)	1.29 (0.86, 1.94)
Ethnicity^d^			
White	Ref.	Ref.	Ref.
Black	1.46 (0.94, 2.25)	1.37 (0.64, 2.88)	1.33 (0.74, 2.36)
Others	0.89 (0.63, 1.25)	0.56 (0.29, 1.05)	1.14 (0.74, 1.74)
BMI	1.03 (1.01, 1.06)**	1.07 (1.01, 1.13)*	1.03 (1.00, 1.06)*
Current smoker	1.01 (0.46, 2.22)	0.47 (0.13, 1.64)	2.08 (0.72, 6.06)
Admission SOFA score	1.36 (1.25, 1.47)***	1.40 (1.20, 1.62)***	1.35 (1.22, 1.50)***
Non-renal SOFA score	1.20 (1.10, 1.32)***	1.24 (1.04, 1.48)*	1.20 (1.07, 1.34)***
Vasopressor use	0.96 (0.66, 1.41)	0.66 (0.35, 1.27)	1.13 (0.68, 1.89)
Ventilation on admission			
Invasive	Ref.	Ref.	Ref.
Non-invasive	0.87 (0.32, 2.35)	–	0.94 (0.32, 2.75)
High-flow nasal cannula	0.39 (0.26, 0.61)***	0.31 (0.12, 0.82)***	0.46 (0.28, 0.76)**
None	0.26 (0.13, 0.51)***	0.18 (0.08, 0.42)**	0.41 (0.11, 1.51)

Clinical biomarkers on admission to ICU	Standardised variables SD (95% CI)		
Baseline creatinine	1.91 (1.44, 2.53)***	2.22 (1.31, 3.75)**	1.72 (1.23, 2.42)**
pH	0.69 (0.58, 0.83)***	0.73 (0.55, 0.98)*	0.64 (0.50, 0.82)***
PaO_2_	1.07 (0.90, 1.27)	0.97 (0.78, 1.20)	0.94 (0.66, 1.33)
Ionised calcium	0.87 (0.74, 1.02)*	1.04 (0.79, 1.39)	0.82 (0.67, 0.99)*
Lactate	1.18 (0.97, 1.44)	1.23 (0.92, 1.82)	1.16 (0.91, 1.49)
Chloride	1.02 (0.87, 1.20)	1.04 (0.76, 1.39)	1.11 (0.89, 1.38)
White blood cells	1.06 (0.90, 1.26)	1.04 (0.71, 1.51)	1.16 (0.95, 1.41)
Neutrophils	1.07 (0.89, 1.30)	1.05 (0.67, 1.64)	1.17 (0.93, 1.47)
Lymphocytes	1.06 (0.89, 1.26)	1.03 (0.76, 1.40)	1.07 (0.87, 1.32)
Haemoglobin	1.05 (0.88, 1.25)	0.94 (0.66, 1.33)	1.02 (0.83, 1.26)
CRP	1.32 (1.10, 1.57) **	1.34 (0.97, 1.87)	1.13 (0.89, 1.41)

Logistic regression was used to examine the relationship between demographic characteristics (exposures) and AKI (outcome). Regression coefficients are represented as odds ratios (95%CI). To allow for comparisons across the clinical biomarkers these variables have been standardised, so that for each variable the mean score was zero with a SD of 1

Models were adjusted for: age, ethnicity, sex, new steroids, remdesivir, any IL-6 antagonists and invasive vs non-invasive ventilation

*BMI* body mass index, *SOFA* Sequential Organ Failure Assessment, *CRP* C-reactive protein

* *p* < 0.05

** *p* < 0.01

*** *p* < 0.001

^a^Also adjusted for wave

^b^Not adjusted for age

^c^Not adjusted for sex

^d^Not adjusted for ethnicity

### KRT rate and risk factors

The KRT rate was lower in wave 2 than wave 1 (13% vs. 32%; *p* < 0.001). The median day of KRT initiation was 3 (IQR 1–6) in wave 1 and 4 (IQR 1–10) in wave 2. The median duration of KRT was 12 (IQR 6–22) days in wave 1 and 11 (IQR 5–23) days in wave 2. The most common indications for KRT in both waves were uremia and oliguria (Additional file 1: Table S3). Multivariate analysis showed that high BMI, invasive MV, high baseline SCr, high lactate, high white blood cell count and high CRP, low pH and low ionised calcium were associated with KRT (Additional file 1: Table S4).

### Risk factors for new AKI and KRT

**Fig. 3:**
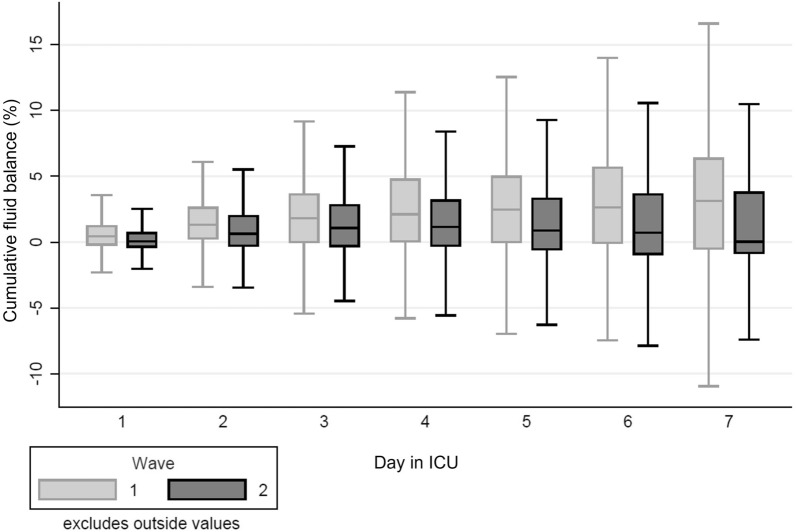
Comparison of daily cumulative fluid balance by COVID-19 wave

**Fig. 4:**
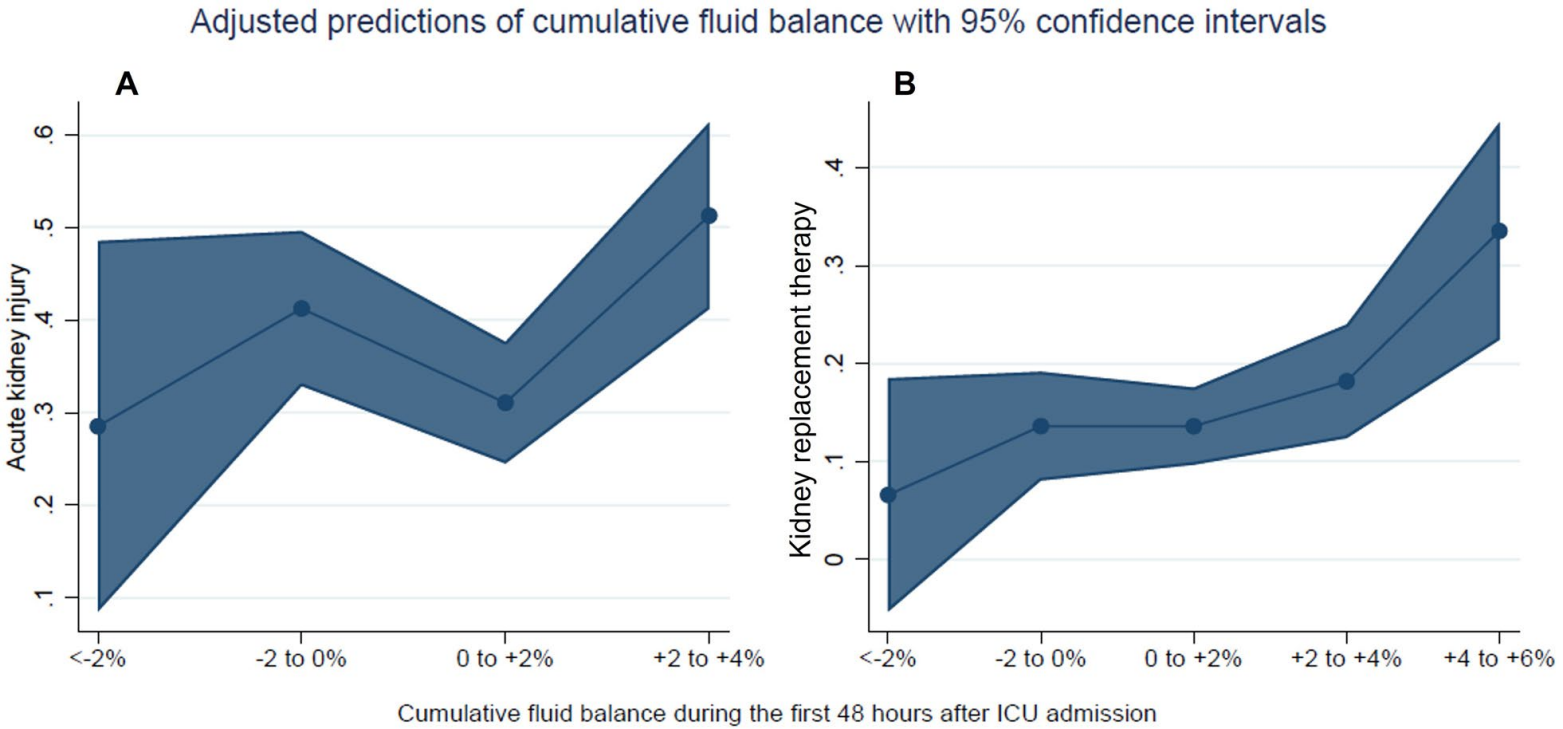
Adjusted risks of developing new-onset AKI (**A**) and KRT (**B**) after 48 h by percentage of 48-h cumulative fluid balance (%). *AKI* acute kidney injury, *KRT* kidney replacement therapy

Compared with wave 2, patients in wave 1 had a higher daily FB despite being administered diuretics more frequently (Fig. 3). The difference was more noticeable in patients admitted from the emergency department and ward but not in those transferred from other hospitals (Additional file 1: Table S5). After adjusting for age, gender, ethnicity, wave, non-renal SOFA, vasopressor use, invasive MV, PEEP levels, diuretics, change in fluid balance and COVID-19 therapies, a positive cumulative FB was independently associated with new AKI and KRT [adjusted OR for 48-h FB > 2% and AKI 2.55 (95% CI 1.46, 4.50); adjusted OR for 48-h FB > 4% and KRT 4.16 (95% CI 2.03, 8.51) (Table 4; Fig. 4).

**Table 4:** Association between cumulative fluid balance during the first 48 h after hospital admission and the development of new AKI and KRT

	Outcome: new AKI [OR (95% CI)] (*n* = 420)^a^	
	Unadjusted	Adjusted
Cumulative FB at 48 h		
< − 2% (*n* = 20)	0.76 (0.26, 2.19)	0.80 (0.26, 2.47)
− 2% to 0% (*n* = 128)	1.40 (0.87, 2.27)	1.57 (0.92, 2.65)
0% to + 2% (*n* = 180)	Reference	Reference
> + 2% (*n* = 92)	3.08 (1.83, 5.20)^***^	2.55 (1.46, 4.50) ^**^
Invasive ventilation	–	1.32 (0.62, 2.84)
Remdesivir	–	0.79 (0.45, 1.38)
Steroids	–	1.49 (0.73, 2.98)
IL-6 antagonists	–	0.78 (0.42, 1.47)
Non-renal SOFA score	–	1.19 (1.04, 1.36)**
Baseline diuretics	–	1.06 (0.63, 1.78)
Max PEEP in first 48 h	–	1.03 (0.97, 1.09)
Vasopressor use	–	0.57 (0.33, 0.98)*
Delta FB between day 1 and 2 [mL/kg]	–	0.99 (0.97, 1.00)

	Outcome: new KRT [OR (95% CI)] (*n* = 648)^b^	
	Unadjusted	Adjusted
Cumulative FB at 48 h		
< − 2% (*n* = 25)	0.26 (0.03, 1.99)	0.44 (0.06, 3.55)
− 2% to 0% (*n* = 161)	0.79 (0.43, 1.44)	1.04 (0.53, 1.89)
0% to + 2% (*n* = 269)	Reference	Reference
+ 2% to + 4% (*n* = 136)	1.70 (0.99, 2.91)	1.57 (0.87, 2.82)
> + 4% (*n* = 57)	3.94 (2.09, 7.45)***	4.16 (2.03, 8.51)***
Invasive ventilation	–	2.70 (1.10, 6.66)*
Remdesivir	–	0.61 (0.29, 1.26)
Steroids	–	3.22 (1.58, 6.54)**
IL-6 antagonists	–	0.84 (0.37, 1.89)
Non-renal SOFA score	–	0.98 (0.86, 1.12)
Baseline diuretics	–	1.14 (0.68, 1.94)
Max PEEP in first 48 h	–	1.00 (0.94, 1.07)
Vasopressor use	–	0.76 (0.45, 1.27)
Delta FB between day 1 and 2 [mL/kg]	–	1.01 (0.99, 1.03)

*AKI* acute kidney injury, *CI* confidence interval, *FB* fluid balance, *KRT* kidney replacement therapy, *IL-6* interleukin-6, *OR* odds ratio, *PEEP* positive end-expiratory pressure, *SOFA* Sequential Organ Failure Assessment

^a ^Adjusted for age, ethnicity, wave, gender, remdesivir, invasive ventilation, new steroids, IL-6 antagonists non-renal SOFA, vasopressors, diuretics, PEEP and change in fluid balance,

^b^Adjusted for age, ethnicity, wave, gender, remdesivir, invasive ventilation, new steroids, IL-6 antagonists non-renal SOFA, vasopressors, diuretics, PEEP and change in fluid balance,

*  *p* < 0.05 **  *p* < 0.01 ***  *p* < 0.001

*n* = 352 excluded; *n* = 74 missing 48-h fluid balance data, *n* = 278 patients were diagnosed with AKI on day 0 and 1

*  *p* < 0.05 **  *p* < 0.01 ***  *p* < 0.001

*n* = 299 excluded; *n* = 74 missing 48-h fluid balance data, *n* = 50 patients received KRT on day 0 and 1

Multivariate analysis showed that treatment with systemic steroids, remdesivir and/or IL-6 antagonists was not associated with AKI development; however, new steroid use was positively associated with KRT after 48 h (adjusted OR 3.18, 95% CI 1.59, 6.36) (Additional file 1: Tables S6, S7).

### Patient and kidney outcomes

The ICU, hospital and 90-day mortality and lengths of stay were similar in both waves (Table 2). AKI was independently associated with 90-day mortality (adjusted HR 2.20, 95% CI 1.16–4.14), adjusted for age, sex, ventilation type, APACHE II score, remdesivir, steroids, IL-6 antagonists, and wave. At hospital discharge, kidney recovery was observed in 89% of AKI patients in wave 2 compared with 83% in wave 1 (*p* < 0.01). Dialysis dependence at discharge was 2% in alive patients in wave 2 compared to 4% in wave 1. At 90 days, 1% of survivors in wave 2 were dialysis dependent compared to 4% in wave 1 (*p* < 0.001). There were no significant changes in SCr or eGFR from baseline, at hospital discharge, and at 90 days between wave 1 and 2 (Additional file 1: Table S8). The proportion of patients with CKD at 90 days was 14% in wave 1 vs. 11% in wave 2. The risk was higher in patients with more severe AKI (Additional file 1: Table S1).

### Sensitivity analysis

We performed a sensitivity analysis for the association between 24-h cumulative FB and risk of new AKI and KRT by excluding patients with AKI or KRT within 24 h of ICU admission. Cumulative FB > 2% was independently associated with KRT receipt after 24 h (adjusted OR 2.14, 95% CI 1.16, 3.94) (Additional file 1: Table S9).

## Discussion

This large analysis of critically ill patients describes changes in AKI epidemiology during the COVID-19 pandemic. The key findings are that fewer ICU patients developed AKI and received KRT in wave 2 despite being older and frailer. When AKI occurred, it was milder, shorter, occurred later and had a better longer-term prognosis. A positive cumulative FB and invasive MV were independent risk factors for new AKI and KRT.

Whilst the improvement may be related to better general management of patients with COVID-19, we also noted that patients had lower baseline SCr values and lower inflammatory markers on admission to ICU (i.e. CRP and ferritin). High CRP is a known risk factor for AKI and KRT [18, 29]. The decline in CRP in patients admitted to ICU during the course of the pandemic might be explained by differences in viral strains or changes in clinical management [17]. Further, the reduced application of invasive MV may have reduced the AKI risk. In the early phase of the pandemic, early intubation was suggested to avoid cross-infection of healthcare workers and to reduce the risk of self-inflicted lung injury [30–35]. This concept changed over time following cumulative data confirming that non-invasive ventilation (NIV) with or without awake prone positioning was effective and safe [36–38].

It has also been suggested that the observed decline in AKI incidence may be due to changes in fluid management, i.e. the abandonment of fluid restriction “to keep the lungs dry” [39]. Our data do not support this hypothesis. In fact, daily cumulative FB was significantly higher in wave 1 than in wave 2 despite using diuretics more often. This is in keeping with results from non-COVID-19 studies showing that higher FB is associated with more AKI and higher need for KRT and longer durations of KRT [27, 40–44].

Another potential explanation for the decline in AKI incidence is the increased use of anti-inflammatory therapies [45]. Landmark studies have reported lower KRT rates in patients who received dexamethasone and tocilizumab [46, 47]. Together with others, we previously reported less AKI progression with steroids [11, 48]. However, a subsequent analysis of a large multi-centre database from the UK did not confirm an association between declining AKI rates and treatment with steroids or remdesivir [19]. In our analysis, steroid use was associated with reduced risk of AKI in univariate analysis but increased risk of KRT in multivariate analysis. These conflicting results suggest that there might be confounding factors that have not been accounted for. For instance, selection bias, interactions between types, doses, and duration of treatments, patient heterogeneity and disease phenotypes [49] may have impacted the risk of AKI and KRT. It should also be acknowledged that the evidence for specific COVID-19 therapies emerged at different times during the pandemic [4]. Although steroids and remdesivir were officially recommended in the UK in May 2020 and IL-6 antagonists were recommended in December 2020, some patients received these medications earlier, for instance, as part of clinical trials [50].

The improved longer-term renal prognosis in the second wave could possibly be explained by a lower proportion of patients with pre-existing CKD. Although the risk of CKD at 90 days declined, it remained relatively high at 11% and as high as 30% in patients with AKI stage 3. At present, little is known about long-term kidney outcomes post-COVID. Hospitalised non-ICU patients with COVID-associated AKI were found to have a greater 6-month decline in eGFR than patients with AKI from other causes [51]. A different study demonstrated an 8.3% GFR decline at 1-year in COVID-19 AKI survivors [52]. Patients with long-COVID without AKI during hospitalisation also had increased risks of ESKD and major adverse kidney events [53]. Inflammatory changes and immune dysfunction following SARS-CoV-2 infection might have contributed [54].

Our study is one of the first describing the changing epidemiology of AKI and KRT in critically ill patients with COVID-19 [17–19, 55]. The strengths are the granular patient-level data, use of both SCr and urine output criteria to define AKI, and inclusion of short- and long-term kidney outcomes up to 90 days. To the best of our knowledge, it is the first study that explores the impact of fluid status on risk of COVID associated AKI in critically ill patients.

Despite these strengths, we acknowledge some limitations. First, this is a single-centre study using only routinely available laboratory and clinical data. About 40% of all patients were transferred from other institutions, either due to clinical needs (e.g. need for extracorporeal membrane oxygenation) or capacity. However, decisions to initiate COVID-19 therapies were based on national guidance. Second, only association but no causality can be implied. Unmeasured confounding factors and treatment bias might not have been accounted for in the models. Third, we did not have data regarding social deprivation status, which could have impacted AKI occurrence. Fourth, we collected detailed FB data, including diuretic use, but data pre-ICU admission were not available for all patients. To assess the association between FB and risks of new AKI and KRT, we focused on patients who had AKI or received KRT 48 h after admission to ICU. Fifth, we followed current consensus recommendations to estimate baseline renal function in patients with missing values. We acknowledge that this may have overestimated baseline function but note that a recent study showed comparable AKI adjudication and outcomes by using either true baseline SCr or SCr results on admission [56]. Sixth, we did not routinely perform urinalysis and did not use novel renal biomarkers during the pandemic and therefore cannot comment on their role in COVID associated AKI. We also did not perform any kidney biopsies and do not know the underlying histopathology. Finally, we acknowledge that both vaccination and evolving virus variants may have modified the AKI and KRT incidences [57]. Unfortunately, complete data regarding patients’ vaccination status and SARS-CoV-2 variants were not available to us. However, during the first two waves, either alpha or delta variant accounted for all ICU infections and milder variants (e.g. omicron) were only present in late 2021.

In summary, although patients in wave 2 were more vulnerable, i.e. older and frailer, we observed reduced rates of AKI and KRT. This decline may be due to changes in inflammatory status along with improved COVID-19 management including lower cumulative FB and changes in respiratory support. Future studies should explore the impact of new variants of the SARS-CoV-2 virus, new immunomodulatory therapies, and vaccination on AKI and KRT requirement and long-term kidney outcomes. Finally, whether current therapies under investigation for long COVID syndrome impact the development of CKD after AKI will need to be investigated.

Our analysis confirms the changing epidemiology of AKI and KRT among critically ill COVID-19 patients with a trend towards less severe and shorter AKI and better long-term prognosis in the second wave. These changes occurred in parallel with decreased initiation of MV, application of lower PEEP and lower daily cumulative FB.

## Supplementary Information

**Additional file 1**: **Figure S1**: Number of patients admitted to ICU, patients with acute kidney injury, and patients who received kidney replacement therapy by month of admission. **Figure S2**: Proportions of patients with acute kidney injury and patients who received kidney replacement therapy by month of admission. **Table S1**: Baseline characteristics, laboratory biomarkers, treatment and outcomes by wave, AKI status and AKI staging. **Table S2**: Unadjusted associations between demographic characteristics and diagnosis of acute kidney injury for all patients and stratified by wave. **Table S3**: Indications for KRT between wave 1 and 2. **Table S4**: Adjusted associations between demographic characteristics and kidney replacement therapy for all patients and stratified by wave. **Table S5**: Comparison of daily cumulative fluid balance (%) by waves and sources of admission. **Table S6**: Unadjusted associations between COVID-19 treatments and AKI or KRT for all patients and stratified by wave. **Table S7**: Treatment and fluid balance for AKI or KRT patients only, stratified by day of diagnosis or KRT and wave of the pandemic. **Table S8**: Changes in serum creatinine and GFR values in alive patients from baseline, hospital discharge, and 90 days after hospital discharge Table S9: Associations between AKI, KRT and 24-hour cumulative fluid balance.

## Data Availability

The datasets used and/or analysed during the current study are available from the corresponding author on reasonable request.

## Abbreviations

AKI: Acute kidney injury
BMI: Body mass index
CI: Confidence interval
CAD: Coronary artery disease
CHF: Congestive heart failure
CKD: Chronic kidney disease
COPD: Chronic obstructive pulmonary disease
COVID-19: Coronavirus disease-19
CRP: C-reactive protein
ECMO: Extracorporeal membrane oxygenation
ED: Emergency department
ESKD: End-stage kidney disease
eGFR: Estimated glomerular filtration rate
FB: Fluid balance
GFR: Glomerular filtration rate
HIV: Human immunodeficiency virus
HR: Hazard ratio
ICU: Intensive care unit
IL: Interleukin
IQR: Interquartile range
KDIGO: Kidney diseases: improving global outcomes
KRT: Kidney replacement therapy
MDRD: Modification of diet in renal disease
MV: Mechanical ventilation
NIV: Non-invasive ventilation
NHS: National Health Service
PEEP: Positive end-expiratory pressure
SARS-CoV-2: Severe acute respiratory syndrome coronavirus 2
SCr: Serum creatinine
SD: Standard deviation
SOFA: Sequential Organ Failure Assessment
UK: United Kingdom
WBC: White blood cells
