# Supplementary material for: Changing epidemiology of acute kidney injury in critically ill patients with COVID-19: a prospective cohort
Source: Ann Intensive Care. 2022 Dec 28;12:118. doi: 10.1186/s13613-022-01094-6 (PMC9794481; doi:10.1186/s13613-022-01094-6)
Supplement: Supplementary file 1 — Additional file 1: Figure S1: Number of patients admitted to ICU, patients with acute kidney injury, and patients who received kidney replacement therapy by month of admission. Figure S2: Proportions of patients with acute kidney injury and patients who received kidney replacement therapy by month of admission. Table S1: Baseline characteristics, laboratory biomarkers, treatment and outcomes by wave, AKI status and AKI staging. Table S2: Unadjusted associations between demographic characteristics and diagnosis of acute kidney injury for all patients and stratified by wave. Table S3: Indications for KRT between wave 1 and 2. Table S4: Adjusted associations between demographic characteristics and kidney replacement therapy for all patients and stratified by wave. Table S5: Comparison of daily cumulative fluid balance (%) by waves and sources of admission. Table S6: Unadjusted associations between COVID-19 treatments and AKI or KRT for all patients and stratified by wave. Table S7: Treatment and fluid balance for AKI or KRT patients only, stratified by day of diagnosis or KRT and wave of the pandemic. Table S8: Changes in serum creatinine and GFR values in alive patients from baseline, hospital discharge, and 90 days after hospital discharge Table S9: Associations between AKI, KRT and 24-hour cumulative fluid balance. [file 13613_2022_1094_MOESM1_ESM.docx]

**Additional File 1**

**Content Page**

**Figure S1: Number of patients admitted to ICU, patients with acute kidney injury, and patients who received kidney replacement therapy by month of admission……………….…………………..…...2**

**Figure S2: Proportions of patients with acute kidney injury and patients who received kidney replacement therapy by month of admission……………………………………………...............................3**

**Table S1: Baseline characteristics, laboratory biomarkers, treatment and outcomes by wave, AKI status and AKI staging…………………………………………………………………………………………………………..…4**

**Table S2: Unadjusted associations between demographic characteristics and diagnosis of acute kidney injury for all patients and stratified by wave…………………………………………………………………8**

**Table S3: Indications for KRT between wave 1 and 2……………………………………………………………..…9**

**Table S4: Adjusted associations between demographic characteristics and kidney replacement therapy for all patients and stratified by wave……………………………………………………………………….10**

**Table S5: Comparison of daily cumulative fluid balance (%) by waves and sources of admission…………………………………………………………………………………….…………………………………………11**

**Table S6: Unadjusted associations between COVID-19 treatments and AKI or KRT for all patients and stratified by wave……………………………………………………………………………….……………………………12**

**Table S7: Treatment and fluid balance for AKI or KRT patients only, stratified by day of diagnosis or KRT and wave of the pandemic…………………………………………………………………..……………………….….13**

**Table S8: Changes in serum creatinine and GFR values in alive patients from baseline, hospital discharge, and 90 days after hospital discharge………………………..…………………………………………..…14**

**Table S9:** **Associations between AKI, KRT and 24-hour cumulative fluid balance……………………...15**

**Figure S1: Number of patients admitted to ICU, patients with acute kidney injury, and patients who received kidney replacement therapy by month of admission at Guy’s & St Thomas’ NHS Foundation Trust**


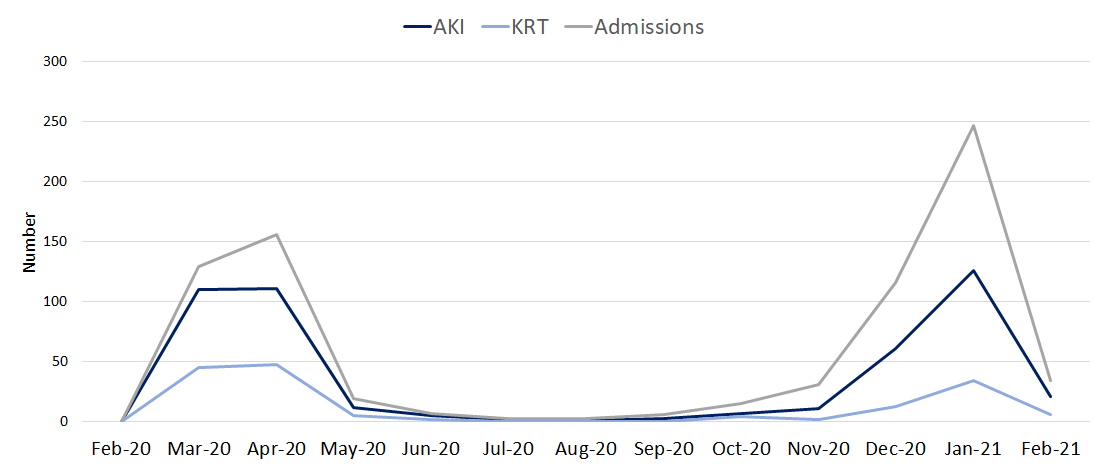


Abbreviations: AKI, acute kidney injury; KRT, kidney replacement therapy

**Figure S2: Proportions of patients with acute kidney injury (AKI) and patients who received kidney replacement therapy (KRT) by month of admission**


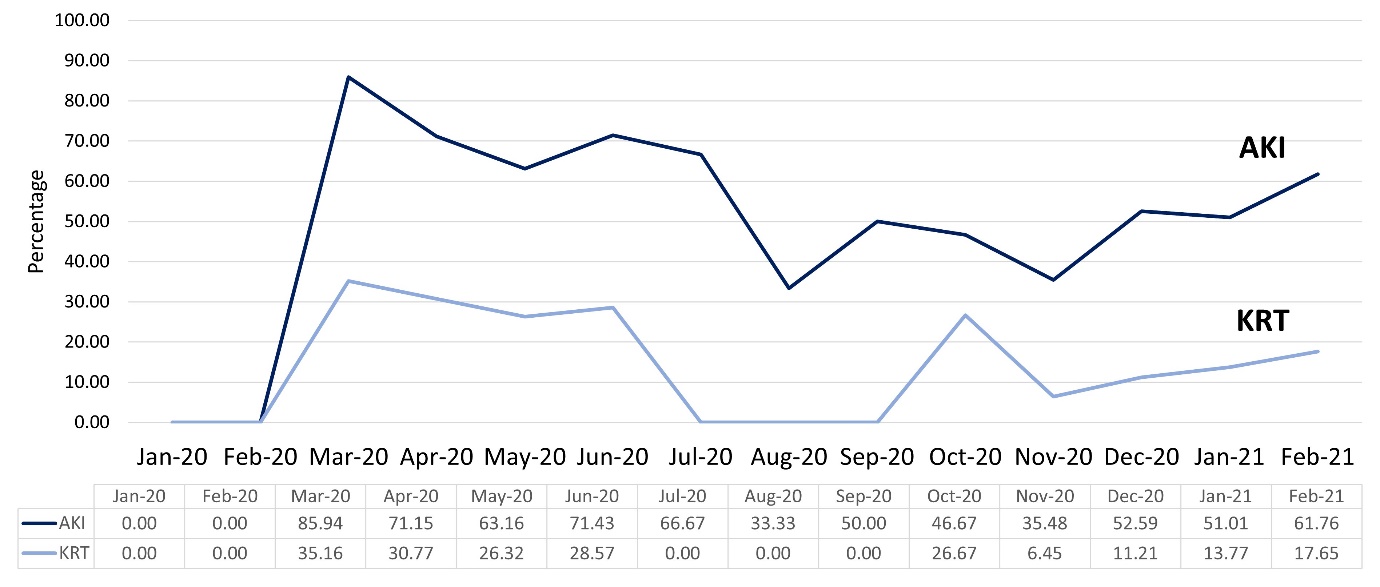


Abbreviations: AKI, acute kidney injury; KRT, kidney replacement therapy

| **Table S1: Baseline characteristics, laboratory biomarkers, treatment and outcomes by wave, AKI status and AKI staging** | | | | | | | | |
| --- | --- | --- | --- | --- | --- | --- | --- | --- |
|  | **Wave 1** | | | | **Wave 2** | | | |
|  | Mean (range) / mean (SD) / N(%) / median (IQR) | | | | | | | |
|  | No AKI  (n=75) | AKI stage 1 (n=64) | AKI stage 2 (n=41) | AKI stage 3 (n=136) | No AKI (n=224) | AKI stage 1 (n=122) | AKI stage 2 (n=40) | AKI stage 3 (n=70) |
| Age (years) | 51.6 (13.8) | 54.1 (16.4) | 57.8 (16.9) | 55.5 (11.5) | 55.9 (12.3) | 57.3 (14.3) | 61.8 (13.9) | 59.0 (12.9) |
| Sex (male) | 51 (68%) | 48 (75%) | 23 (56%) | 100 (73%) | 151 (67%) | 83 (68%) | 22 (55%) | 41 (59%) |
| Ethnicity  White  Black  Other  Not available | 26 (35%)  16 (21%) 19 (25%)  14 (19%) | 25 (39%)  21 (33%)  11 (17%)  7 (11%) | 16 (39%)  12 (29%)  9 (20%)  5 (12%) | 53 (39%)  41 (30%)  13 (10%)  29 (21%) | 95 (42%)  34 (15%)  42 (19%) 53 (24%) | 44 (36%)  14 (11%)  28 (23%)  36 (30%) | 13 (33%)  8 (20%)  9 (23%)  10 (25%) | 27 (39%)  16 (23%)  7 (10%)  20 (29%) |
| BMI | 27.6 (23.3, 32.0) | 27.8 (24.2, 32.9) | 28.7 (24.7, 32.0) | 29.4 (26.1, 34.9) | 29.0 (26.0, 34.6) | 29.1 (26.0, 38.4) | 30.3 (25.0, 35.7) | 31.2 (25.7, 27.1) |
| Days of symptoms | 10 (7, 15) | 10 (5, 13) | 8 (4, 12) | 9 (7, 12) | 10 (7, 14) | 8 (5, 12) | 10 (6, 11) | 6 (4, 11) |
| SOFA score | 4 (2, 6) | 5 (4, 6) | 5 (4, 7) | 6 (4, 8) | 3 (2, 5) | 5 (3, 7) | 5 (4, 6) | 6 (4, 9) |
| Comorbidities  Diabetes | 12 (16%) | 22 (34%) | 11 (26%) | 51 (38%) | 54 (24%) | 44 (36%) | 13 (33%) | 26 (37%) |
| Asthma | 14 (19%) | 4 (6%) | 5 (12%) | 25 (18%) | 45 (20%) | 13 (11%) | 7 (18%) | 4 (6%) |
| Hypertension | 19 (25%) | 23 (36%) | 17 (41%) | 68 (50%) | 83 (37%) | 68 (56%) | 19 (48%) | 35 (50%) |
| CAD | 2 (3%) | 4 (6%) | 4 (10%) | 6 (4%) | 19 (8%) | 14 (11%) | 3 (7%) | 5 (7%) |
| CHF | 2 (3%) | 2 (3%) | 5 (12%) | 5 (4%) | 6 (3%) | 2 (3%) | 5 (12%) | 5 (4%) |
| Atrial fibrillation/Atrial flutter | 1 (1%) | 2 (3%) | 5 (12%) | 3 (2%) | 6 (3%) | 5 (4%) | - | 1 (1%) |
| COPD | - | 3 (5%) | 3 (7%) | 6 (4%) | 18 (8%) | 8 (7%) | 6 (15%) | 3 (4%) |
| Chronic kidney disease | 1 (1%) | 8 (13%) | 5 (10%) | 9 (7%) | 8 (4%) | 9 (7%) | 3 (8%) | 13 (19%) |
| Chronic liver disease | 1 (1%) | 1 (2%) | 2 (5%) | 8 (6%) | 7 (3%) | 7 (6%) | - | 3 (4%) |
| HIV infection | 3 (4%) | 3 (5%) | - | 1 (1%) | 4 (2%) | 2 (2%) | 1 (2%) | 2 (3%) |
| Malignancy | 3 (4%) | 3 (5%) | 6 (15%) | 2 (1%) | 12 (5%) | 6 (5%) | 2 (5%) | 3 (4%) |

|  | **Wave 1** | | | | **Wave 2** | | | |
| --- | --- | --- | --- | --- | --- | --- | --- | --- |
|  | Mean (range)/ mean (SD)/ N(%)/ median (IQR) | | | | | | | |
|  | No AKI  (n=75) | AKI stage 1 (n=64) | AKI stage 2 (n=41) | AKI stage 3 (n=136) | No AKI (n=224) | AKI stage 1 (n=122) | AKI stage 2 (n=40) | AKI stage 3 (n=70) |
| **Baseline laboratory parameters and organ support on Day 1** | | | | | | | | |
| Respiratory support  Invasive ventilation  Non-invasive ventilation  High-flow nasal cannula  None | 48 (64%)  10 (13%)  10 (13%)  17 (23%) | 50 (78%)  1 (2%)  6 (9%)  7 (11%) | 33 (80%)  2 (5%)  2(5%)  4 (10%) | 123 (90%)  1 (1%)  6 (4%)  6 (4%) | 115 (51%)  8 (4%)  94 (42%)  7 (3%) | 85 (70%)  3 (3%)  31 (25%)  2 (2%) | 27 (68%)  1 (2%)  11 (28%)  1 (2%) | 51 (72%)  3 (4%)  15 (21%)  1 (1%) |
| No. of vasopressors  0  1  2  3 | 52 (69%)  20 (27%)  2 (3%)  1 (1%) | 42 (66%)  22 (34%)  -  - | 22 (54%)  16 (39%)  3 (7%)  - | 67 (49%)  63 (46%)  6 (4%)  - | 178 (79%)  43 (19%)  3 (1%)  - | 74 (61%)  47 (39%)  1 (1%)  - | 29 (73%)  11 (28%)  -  - | 39 (56%)  29 (41%)  2 (3%)  - |
| **Clinical biomarkers** |  |  |  |  |  |  |  |  |
| Baseline serum creatinine (mmol/L) | 73 (59, 85) | 83 (70, 95) | 81 (70, 99) | 88 (70, 100) | 73 (61, 88) | 78 (65, 95) | 78 (71, 88) | 88 (71, 98) |
| pH | 7.4 (7.3, 7.5) | 7.4 (7.3, 7.4) | 7.3 (7.3, 7.4) | 7.4 (7.2, 7.4) | 7.4 (7.4, 7.5) | 7.4 (7.4, 7.5) | 7.4 (7.3, 7.5) | 7.3 (7.3, 7.4) |
| PaO_2_ | 9.9 (8.6, 12.4) | 10.2 (8.7, 14.4) | 10.4 (8.6, 12.8) | 10.0 (8.8, 12.6) | 8.3 (7.3, 9.5) | 8.3 (7.2, 9.6) | 8.1 (7.2, 9.4) | 8.5 (7.3, 9.8) |
| WBC (10^9/L) | 8.6 (6.3, 12.6) | 8.8 (5.9, 12.7) | 9.2 (6.7, 12.5) | 8.7 (6.4, 11.9) | 8.7 (6.0, 12.8) | 10.1 (7.1, 13.4) | 9.3 (7.2, 13.3) | 12.5 (8.0, 17.2) |
| Neutrophils (10^9/L) | 7.0 (4.7, 10.6) | 7.7 (4.3, 11.7) | 7.6 (5.5, 9.9) | 7.6 (5.1, 10.2) | 7.7 (4.7, 11.3) | 8.6 (6.0, 12.2) | 8.4 (6.1, 11.8) | 10.6 (6.9, 15.4) |
| Lymphocytes (10^9/L) | 0.8 (0.5, 1.1) | 0.6 (0.4, 0.9) | 0.9 (0.5, 1.3) | 0.7 (0.5, 1.0) | 0.7 (0.4, 0.9) | 0.6 (0.4, 0.9) | 0.7 (0.4, 1.0) | 0.7 (0.4, 1.2) |
| Haemoglobin (g/L) | 118.6 (21.5) | 119.6 (22.2) | 112.0 (20.1) | 117.3 (21.2) | 119.8 (20.7) | 118 (26.2) | 120 (22.2) | 110.9 (28.9) |
| Ferritin (µg/L) | 923 (660, 1429) | 1164 (611, 2014) | 1118 (404, 2057) | 1481 (757, 2870) | 1053 (541, 1799) | 942 (482, 1818) | 903 (410, 1603) | 1130 (701, 2605) |
| D-dimer (mg/L) | 1.35 (0.65, 4.36) | 1.3 (0.70, 3.96) | 4.6 (0.96, 10.30) | 1.97 (0.80, 8.40) | 1.56 (0.78, 5.55) | 2.5 (1.02, 7.65) | 1.66 (0.78, 8.50) | 3.0 (1.62, 11.93) |

|  | **Wave 1** | | | | **Wave 2** | | | |
| --- | --- | --- | --- | --- | --- | --- | --- | --- |
|  | Mean (range)/ mean (SD)/ N(%)/ median (IQR) | | | | | | | |
| CRP (mg/L) | 131 (51, 291) | 168 (61, 303) | 149 (94, 257) | 202 (121, 321) | 80 (41, 154) | 96 (41, 176) | 116 (56, 197) | 92 (57, 207) |
|  | No AKI  (n=75) | AKI stage 1 (n=64) | AKI stage 2 (n=41) | AKI stage 3 (n=136) | No AKI (n=224) | AKI stage 1 (n=122) | AKI stage 2 (n=40) | AKI stage 3 (n=70) |
| PaO_2_/FiO_2_ ratio | 21.9 (16.2, 29.0) | 20.0 (14.4, 26.7) | 17.2 (12.2, 24.3) | 16.4 (12.4, 20.9) | 13.9 (11.0, 17.9) | 12.7 (10.0, 17.4) | 12.2 (9.6, 15.9) | 12.6 (9.1, 18.1) |
| **Treatments** |  |  |  |  |  |  |  |  |
| New systemic steroids | 37 (49%) | 32 (50%) | 23 (56%) | 94 (69%) | 220 (98%) | 121 (99%) | 40 (100%) | 69 (99%) |
| Remdesivir | 9 (12%) | 3 (5%) | 5 (12%) | 2 (12%) | 115 (51%) | 42 (34%) | 15 (38%) | 17 (24%) |
| IL-6 antagonists | 1 (1%) | - | - | 2 (1%) | 67 (29%) | 31 (25%) | 10 (25%) | 14 (20%) |
| **Patient outcomes** | | | | | | | | |
| Mechanical ventilation during admission | 51 (68%) | 53 (83%) | 35 (85%) | 130 (96%) | 127 (57%) | 95 (77%) | 32 (80%) | 65 (93%) |
| ICU mortality | 9 (12%) | 10 (16%) | 11 (27%) | 59 (43%) | 31 (14%) | 34 (28%) | 6 (15%) | 34 (49%) |
| Hospital mortality | 9 (12%) | 10 (16%) | 12 (29%) | 61 (45%) | 35 (16%)* | 36 (30%) | 7 (18%) | 35 (50%)* |
| ICU length of stay | 7 (3, 22) | 12.5 (5.5, 30.5) | 16 (6, 27) | 17 (10, 31) | 9 (5, 26) | 14 (7, 30) | 14 (10, 28) | 19 (11, 30) |
| Hospital length of stay | 15 (8, 32) | 20 (13, 40) | 25 (8, 41) | 24 (12, 47) | 19 (10, 44) | 21 (12, 45) | 28 (18, 47) | 22 (14, 54) |
| **Kidney outcomes** | | | | | | | | |
| Dialysis dependence at 30 days | 1 (1%) | 1 (2%) | 1 (2%) | 49 (36%) | 1 (0.5%) | 3 (2%) | - | 35 (51%) |
| Kidney recovery at hospital discharge^2^ | 66 (100%) | 54 (100%) | 29 (100%) | 49 (65%) | 187 (99%) | 84 (98%) | 30 (90%) | 22 (65%) |
| Dialysis dependence at discharge^2^ | 0/66 | 0/54 | 0/29 | 8/75 (11%) | 0/188 | 0/86 | 0/33 | 7/27 (21%) |
| **90-day outcomes after day of discharge** | | | | | | | | |
| Dialysis dependence^3^ | 3 (4%) | 1 (2%) | - | 5 (7%) | - | - | - | 2 (6%) |
| eGFR (ml/min/1.73m^2^) | 100 (79, 117) | 96 (81, 115) | 87 (65, 109) | 77 (54, 105) | 98 (81, 109) | 93 (77, 106) | 93 (66, 106) | 80 (33, 108) |
| CKD | 1 (2%) | 2 (4%) | 5 (19%) | 19 (29%) | 9 (8%) | 3 (7%) | 4 (17%) | 7 (30%) |
| 90-day mortality | 10 (13%) | 10 (16%) | 12 (29%) | 61 (45%) | 40 (18%) | 37 (30%) | 8 (20%) | 35 (50%) |
| Abbreviations: SD, standard deviation; IQR, interquartile range; N, number; BMI, body mass index; SOFA, Sequential Organ Failure Assessment; APACHE II, Acute Physiologic and Chronic Health Evaluation II; CAD, coronary artery disease; CHF, congestive heart failure; COPD, chronic obstructive pulmonary disease; HIV, human immunodeficiency virus; CRP, c-reactive protein; AKI, acute kidney injury; ICU, intensive care unit; WBC, white blood cells; CKD, chronic kidney disease; eGFR, estimated glomerular filtration rate; IL-6, interleukin 6  Binary and categorical variables are presented using counts and percentages. The distribution of continuous variables was assessed using coefficients of skewness and then summarized by mean and standard deviation or median and interquartile range where appropriate.  ^1^ in survivors ^$^Data not available in 25 patients in wave 1 and 7 patients in wave 2  ^2^ 2 patients without AKI and 1 patient with AKI stage 3 still hospitalised until the end of the study date | | | | | | | | |

| **Table S2: Unadjusted associations between demographic characteristics and diagnosis of acute kidney injury for all patients and stratified by wave** | | | |
| --- | --- | --- | --- |
|  | **All participants (n=772)** | **Wave 1 (n=316)** | **Wave 2 (n=456)** |
|  | Odds ratios (95% CI) | | |
| Age | 1.01 (1.00, 1.02)* | 1.02 (1.00, 1.04)* | 1.02 (1.00, 1.03)* |
| Sex (male) | 1.02 (0.75, 1.40) | 0.87 (0.50, 1.52) | 1.21 (0.83, 1.79) |
| Ethnicity  White  Black  Others  Not available | Ref  1.52 (1.01, 2.28)*  0.85 (0.56, 1.27)  1.09 (0.74, 1.59) | Ref  1.28 (0.64, 2.56)  0.47 (0.23, 0.95)*  0.81 (0.38, 1.71) | Ref  1.26 (0.73, 2.19)  1.18 (0.71, 1.98)  1.41 (0.88, 2.24) |
| BMI | 1.01 (0.99, 1.04) | 1.06 (1.00, 1.11)* | 1.02 (0.99, 1.05) |
| Respiratory support on admission  Invasive ventilation  Non-invasive ventilation  High-flow nasal cannula  None | Ref  0.66 (0.27, 1.65)  0.30 (0.21, 0.43)***  0.38 (0.21, 0.71)** | Ref  -  0.33 (0.14, 0.78)**  0.23 (0.11, 0.49)*** | Ref  0.71 (0.26, 1.93)  0.43 (0.28, 0.64)***  0.40 (0.12, 1.41) |
| **Laboratory parameters** | Standardised variables SD (95% CI) | | |
| Baseline creatinine | 1.76 (1.39, 2.22)*** | 2.29 (1.45, 3.61)*** | 1.50 (1.14, 1.98)** |
| pH | 0.60 (0.50, 0.71)*** | 0.73 (0.55, 0.97)* | 0.59 (0.47, 0.73)*** |
| PaO_2_ | 1.29 (1.08, 1.54)** | 0.99 (0.80, 1.22) | 1.07 (0.78, 1.47) |
| Ionised calcium | 0.83 (0.71, 0.96)* | 0.94 (0.72, 1.24) | 0.81 (0.66, 0.98)* |
| Lactate | 1.17 (0.98, 1.39) | 1.23 (0.88, 1.72) | 1.15 (0.91, 1.45) |
| Chloride | 1.01 (0.87, 1.18) | 1.08 (0.82, 1.41) | 1.21 (0.99, 1.49) |
| White blood cells | 1.17 (1.00, 1.37)* | 1.00 (0.72, 1.40) | 1.31 (1.08, 1.58)** |
| Neutrophils | 1.19 (0.99, 1.42) | 1.02 (0.69, 1.51) | 1.36 (1.08, 1.70)** |
| Lymphocytes | 1.09 (0.93, 1.28) | 1.00 (0.75, 1.33) | 1.08 (0.89, 1.30) |
| Haemoglobin | 0.88 (0.76, 1.02) | 0.92 (0.68, 1.24) | 0.86 (0.72, 1.03) |
| CRP | 1.48 (1.25, 1.75) ** | 1.55 (1.15, 2.10)** | 1.17 (0.95, 1.46) |
| Non-renal SOFA score | 1.28 (1.19, 1.37)*** | 1.28 (1.12, 1.43)*** | 1.25 (1.15, 1.36)*** |
| Baseline diuretics | 1.64 (1.17, 2.30_** | 1.82 (1.02, 3.26)* | 1.18 (0.75, 2.86) |
| Max PEEP | 1.09 (1.06, 1.12)*** | 1.10 (1.05, 1.16)*** | 1.07 (1.03, 1.11)*** |
| Vasopressor use | 2.09 (1.55, 2.82)*** | 1.53 (0.85, 2.48) | 2.20 (1.48, 3.26)*** |
| Fluid balance ml/kg (day 1 and 2) | 0.99 (0.99, 1.00) | 1.00 (0.98, 1.02) | 0.99 (0.98, 1.00) |
| Logistic regression was used to examine the relationship between demographic characteristics (exposures) and AKI (outcome). Regression coefficients are represented as odds ratios (95%CI). To allow for comparisons across the clinical biomarkers these variables have been standardised, so that for each variable the mean score was zero with a SD of 1.  * <0.05 **<0.01 ***<0.001. | | | |

Abbreviations: SD, standard deviation; IQR, interquartile range; N, number; BMI, body mass index; SOFA, Sequential Organ Failure Assessment; CRP, c-reactive protein; AKI, acute kidney injury; PEEP = positive end-expiratory pressure

| **Table S3: Indications for KRT between wave 1 and 2** | | |
| --- | --- | --- |
|  | **Wave 1 (n=100)** | **Wave 2 (n=60)** |
| Hyperkalaemia | 37 (37%) | 13 (21.7%) |
| Oliguria | 67 (67%) | 32 (53.3%) |
| Acidosis | 25 (25%) | 11 (18.3%) |
| High urea/uraemic symptoms | 73 (73%) | 35 (58.3%) |
| Pulmonary oedema/fluid overload | 15 (15%) | 11 (18.3%) |
| Others | 10 (10%)^1^ | 1 (1.7%)^2^ |
| ^1^Rhabdomyolysis (n=4), Hypernatraemia (n=5), Obstructive uropathy (n=1)  ^2^Rhabdomyolysis (n=1)  N.B: Options are not mutually exclusive. | | |

Abbreviations: KRT, kidney replacement therapy

| **Table S4: Adjusted associations between demographic characteristics and kidney replacement therapy for all patients and stratified by wave** | | | |
| --- | --- | --- | --- |
|  | **All participants^1^ (n=772)** | **Wave 1 (n=316)** | **Wave 2 (n=456)** |
|  | Odds ratios (95% CI) | | |
| Age^2^ | 1.01 (0.99, 1.02) | 1.01 (0.99, 1.03) | 1.02 (1.00, 1.04) |
| Sex (male)^3^ | 0.72 (0.48, 1.09) | 0.48 (0.27, 0.89)* | 1.24 (0.69, 2.23) |
| Ethnicity^4^  White  Black  Others | Ref  1.39 (0.86, 2.25)  0.85 (0.56, 1.30) | Ref  1.37 (0.73, 2.57)  1.04 (0.56, 1.91) | Ref  1.09 (0.48, 2.50)  0.79 (0.42, 1.48) |
| BMI | 1.04 (1.01, 1.07)** | 1.06 (1.02, 1.11)** | 1.04 (1.00, 1.08) |
| Current smoker | 1.24 (0.49, 3.11) | 1.16 (0.31, 4.32) | 1.54 (0.40, 5.92) |
| Admission non-renal SOFA score | 1.02 (0.92, 1.13) | 0.66 (0.83, 1.13) | 1.05 (0.90, 1.22) |
| Vasopressor use | 1.22 (0.82, 1.82) | 1.08 (0.63, 1.86) | 1.60 (0.84, 3.03) |
| Respiratory support on admission  Invasive ventilation  Non-invasive ventilation  High-flow nasal cannula  None | Ref  0.89 (0.24, 3.34)  0.38 (0.19, 0.79)**  0.07 (0.01, 0.51)** | Ref  0.90 (0.08, 10.17)  0.31 (0.08, 1.16)  0.06 (0.01, 0.47)** | Ref  1.15 (0.23, 5.85)  0.52 (0.20, 1.32)  - |
| **Laboratory parameters** | Standardised variables SD (95% CI) | | |
| Baseline creatinine | 2.06 (1.58, 2.68)*** | 1.82 (1.28, 2.58)** | 2.40 (1.59, 3.62)*** |
| pH | 0.59 (0.49, 0.72)*** | 0.70 (0.54, 0.92)* | 0.43 (0.31, 0.60)*** |
| PaO_2_ | 1.02 (0.85, 1.22) | 0.91 (0.72, 1.16) | 1.03 (0.65, 1.64) |
| Ionised calcium | 0.67 (0.55, 0.83)*** | 0.86 (0.64, 1.16) | 0.56 (0.41, 0.75)*** |
| Lactate | 1.16 (0.99, 1.37) | 1.05 (0.83, 1.32) | 1.36 (1.02, 1.82)* |
| Chloride | 0.95 (0.79, 1.14) | 1.24 (0.95, 1.62) | 0.87 (0.64, 1.19) |
| White blood cells | 1.22 (1.01, 1.47)* | 1.15 (0.82, 1.62) | 1.43 (1.11, 1.86)** |
| Neutrophils | 1.08 (0.90, 1.29) | 1.21 (0.81, 1.82) | 1.14 (0.92, 1.42) |
| Lymphocytes | 0.97 (0.81, 1.16) | 0.89 (0.66, 1.18) | 1.02 (0.79, 1.32) |
| Haemoglobin | 0.84 (0.68, 1.04) | 0.81 (0.59, 1.12) | 0.76 (0.55, 1.04) |
| CRP | 1.60 (1.33, 1.94)*** | 1.74 (1.30, 2.32)*** | 1.13 (0.83, 1.54) |
| Logistic regression was used to examine the relationship between demographic characteristics (exposures) and KRT (outcome). Regression coefficients are represented as odds ratios (95%CI). To allow for comparisons across the clinical biomarkers these variables have been standardised, so that for each variable the mean score was zero with a SD of 1.  * <0.05 **<0.01 ***<0.001.  Models were adjusted for: age, ethnicity, sex, new steroids, remdesivir, IL6 antagonists and invasive vs non-invasive ventilation.  ^1^ also adjusted for wave. ^2^ not adjusted for age, ^3^ not adjusted for sex, ^4^ not adjusted for ethnicity. | | | |

Abbreviations: SD, standard deviation; CI, confidence interval; BMI, body mass index; CRP, c-reactive protein

| **Table S5: Comparison of daily cumulative fluid balance (%) by waves and sources of admission** | | | | |
| --- | --- | --- | --- | --- |
| **Source of admission/Day after ICU admission** | **No.** | **Wave 1 (n=316)** | **No.** | **Wave 2 (n=456)** |
| Mean (SD) | | | | |
| **Emergency department** | | | | |
| Day 1 | 85 | 0.89 (1.75) | 116 | 0.44 (1.31) |
| Day 2 | 83 | 2.10 (2.72) | 115 | 1.36 (2.02) |
| Day 3 | 79 | 3.17 (3.79) | 114 | 1.92 (2.50) |
| Day 4 | 73 | 3.79 (4.40) | 113 | 1.99 (2.97) |
| Day 5 | 68 | 4.43 (5.12) | 114 | 2.08 (3.33) |
| Day 6 | 64 | 4.93 (5.57) | 114 | 2.14 (3.85) |
| Day 7 | 63 | 5.43 (5.84) | 113 | 2.29 (4.03) |
| **Ward** | | | |  |
| Day 1 | 103 | 0.53 (1.18) | 107 | 0.14 (1.11) |
| Day 2 | 103 | 1.29 (1.95) | 107 | 0.44 (1.94) |
| Day 3 | 98 | 1.96 (2.72) | 107 | 0.39 (2.75) |
| Day 4 | 93 | 2.46 (3.30) | 107 | 0.40 (3.08) |
| Day 5 | 85 | 3.00 (3.68) | 107 | 0.45 (3.54) |
| Day 6 | 81 | 3.52 (4.03) | 107 | 0.58 (3.88) |
| Day 7 | 71 | 4.14 (4.41) | 107 | 0.69 (4.38) |
| **Transfer from other centres** | | | | |
| Day 1 | 108 | 0.38 (1.26) | 176 | 0.25 (1.08) |
| Day 2 | 108 | 1.28 (2.25) | 176 | 0.96 (1.79) |
| Day 3 | 106 | 1.44 (2.80) | 176 | 1.43 (2.47) |
| Day 4 | 102 | 1.61 (3.65) | 176 | 1.64 (3.14) |
| Day 5 | 101 | 1.58 (4.35) | 176 | 1.45 (3.62) |
| Day 6 | 98 | 1.32 (4.88) | 176 | 1.30 (4.09) |
| Day 7 | 95 | 1.01 (5.06) | 175 | 0.97 (4.51) |
| **Others** | | | | |
| Day 1 | 3 | 1.51 (1.76) | 3 | 0.30 (0.16) |
| Day 2 | 3 | 1.90 (1.59) | 3 | -0.22 (2.00) |
| Day 3 | 3 | 1.93 (1.60) | 3 | -0.35 (0.87) |
| Day 4 | 3 | 2.70 (2.70) | 3 | -0.25 (0.83) |
| Day 5 | 3 | 2.41 (2.66) | 3 | 0 |
| Day 6 | 3 | 3.12 (2.61) | 3 | 0 |
| Day 7 | 2 | 1.87 (2.89) | 3 | 0 |

| **Table S6: Unadjusted associations between COVID-19 treatments and AKI or KRT for all patients and stratified by wave** | | | | | | |
| --- | --- | --- | --- | --- | --- | --- |
| ***Supplementary Table 6a: Unadjusted associations between treatments and AKI for all patients and stratified by wave*** | | | | | | |
|  | **All participants (n=772)** | | **Wave 1 (n=316)** | | | **Wave 2 (n=456)** |
|  | Odds ratios (95% CI) | | | | | |
| New steroids | 0.66 (0.44, 0.98)* | | 1.66 (0.99, 2.80) | | | 2.09 (0.38, 11.53) |
| Remdesivir | 0.30 (0.22, 0.42)*** | | 0.32 (0.12, 0.81)* | | | 0.44 (0.30, 0.64)*** |
| IL-6 antagonists | 0.46 (0.32, 0.69)*** | | 0.62 (0.06, 6.92) | | | 0.73 (0.48, 1.10) |
| Logistic regression was used to examine the relationship between treatments (exposures) and AKI (outcome). Regression coefficients are represented as odds ratios (95% CI).  * <0.05 **<0.01 ***<0.001. | | | | | | |
| ***Supplementary Table 6b: Unadjusted associations between treatments and KRT for all patients and stratified by wave*** | | | | | | |
|  | | **All participants (n=772)** | | **Wave 1 (n=316)** | | **Wave 2 (n=456)** |
|  | | Odds ratios (95% CI) | | | | |
| New steroids | | 1.07 (0.67, 1.70) | | 2.64 (1.57, 4.45)^***^ | | 0.75 (0.09, 6.57) |
| Remdesivir | | 0.22 (0.13, 0.39)*** | | 0.24 (0.05, 1.05) | | 0.35 (0.18, 0.66) ** |
| IL-6 antagonists | | 0.39 (0.22, 0.72)*** | | - | | 0.73 (0.38, 1.40) |
| Logistic regression was used to examine the relationship between treatments (exposures) and KRT (outcome). Regression coefficients are represented as odds ratios (95% CI).  * <0.05 **<0.01 ***<0.001. | | | | | | |
| ***Supplementary Table 6c: Unadjusted associations between steroid dose and KRT for all patients and stratified by wave*** | | | | | | |
|  | | **All participants (n=772)** | | **Wave 1 (n=316)** | **Wave 2 (n=456)** | |
|  | | Odds ratios (95% CI) | | | | |
| No steroids (n=136) | | Ref | | Ref | | Ref |
| Low dose (n=332) | | 1.10 (0.66, 1.80) | | 3.15 (1.80, 5.53)*** | | 0.49 (0.05, 4.39) |
| High dose (n=304) | | 1.03 (0.62, 1.71) | | 1.91 (0.98, 3.72) | | 1.02 (0.12, 8.97) |
| Logistic regression was used to examine the relationship between dose of steroids (exposures) and KRT (outcome). Regression coefficients are represented as odds ratios (95% CI).  * <0.05 **<0.01 ***<0.001. | | | | | | |

Abbreviations: AKI, acute kidney injury; CI, confidence interval; IL-6, interleukin 6; KRT, kidney replacement therapy

| **Table S7: Treatment and fluid balance for AKI or KRT patients only, stratified by day of diagnosis or KRT and wave of the pandemic** | | | | | | |
| --- | --- | --- | --- | --- | --- | --- |
|  | **Wave 1 (n=316)** | | | **Wave 2 (n=456)** | | |
|  | **No AKI (n=75)** | **AKI (day 0/1) (n=160)** | **AKI (day 2+) (n=81)** | **No AKI (n=224)** | **AKI (day 0/1) (n=139)** | **AKI (day 2+) (n=93)** |
| Steroids | 37 (49%) | 97 (61%) | 52 (64%) | 220 (98%) | 137 (98%) | 93 (100%) |
| IL-6 antagonists | 1 (1%) | 2 (1%) | - | 67 (30%) | 29 (21%) | 26 (28%) |
| Remdesivir | 9 (12%) | 7 (4%)* | 3 (4%) | 115 (51%) | 34 (24%)*** | 40 (43%) |
| Percentage of FB at 48hr;  median (IQR) | 0.45  (-0.60, 1.68) | 1.41 (0.41, 2.98)*** | 1.44 (-0.18, 2.87)** | 0.36 (-0.55, 1.33) | 1.47 (0.13, 3.15)*** | 0.59 (-0.45, 1.77) |
| b-coefficient (95% CI)^$^ | Reference | 1.26 (0.62, 1.91)*** | 0.77 (0.03, 1.50)* | Reference | 1.30 (0.89, 1.72)*** | 0.19 (-0.28, 0.67) |
|  | **No KRT (n=216)** | **KRT (day 0/1) (n=31)** | **KRT (day 2+) (n=69)** | **No KRT (n=396)** | **KRT (day 0/1) (n=20)** | **KRT (day 2+) (n=40)** |
| Steroids | 112 (52%) | 19 (61%) | 55 (80%)*** | 391 (99%) | 19 (95%) | 40 (100%) |
| IL-6 antagonists | 3 (1%) | - | - | 109 (28%) | 3 (15%) | 10 (25%) |
| Remdesivir | 17 (8%) | - | 2 (3%) | 176 (44%) | 2 (10%)** | 11 (28%)* |
| Percentage of FB at 48hr;  median (IQR) | 1.22 (-0.06, 2.32) | 0.87 (0.35, 2.23) | 2.25 (1.04, 4.22)*** | 0.54 (-0.35, 1.78) | 1.89 (-0.66, 4.16)* | 1.18 (0.31, 2.82)* |
| b-coefficient (95% CI)^$^ | Reference | 0.30 (-0.56, 1.17) | 1.43 (0.81, 2.05)*** | Reference | 1.12 (0.26, 1.99)* | 0.80 (0.17, 1.44)* |
| P values are compared between AKI and KRT vs no AKI or no KRT.  * <0.05 **<0.01 ***<0.001.  ^$^Beta-coefficient of fluid balance | | | | | | |

Abbreviations: AKI, acute kidney injury; CI, confidence interval; FB, fluid balance; IL-6, interleukin 6; IQR, interquartile range; KRT, kidney replacement therapy

| **Table S8: Changes in serum creatinine and GFR values in alive patients from baseline, hospital discharge, and 90 days after hospital discharge** | | | | | | | | | |
| --- | --- | --- | --- | --- | --- | --- | --- | --- | --- |
| ***Supplementary Table 8a: Changes in creatinine levels (alive patients only)*** | | | | | | | | | |
|  | | **Wave 1** | | | | **Wave 2** | | | |
| Difference in creatinine values from baseline to hospital discharge (mmol/L) | | -11 (-22, 5) | | | | -8 (-21, 5) | | | |
| Percentage of change in creatinine values from baseline to hospital discharge (%) | | -14.4 (-26.7, 8.7) | | | | -9.7 (-25.8, 6.5) | | | |
| Difference in creatinine values from baseline to 90 days (mmol/L) | | -6 (-20, 9) | | | | -6 (-17, 5) | | | |
| Percentage of change in creatinine values from baseline to 90 days (%) | | -7.5 (-24, 10.6) | | | | -7.8 (-19.5, 8.1) | | | |
| - No significant differences in creatinine between wave 1 and 2. | | | | | | | | | |
| ***Supplementary Table 8b: Changes in eGFR (alive patients only)*** | | | | | | | | | |
|  | **Wave 1** | | | | **Wave 2** | | | | P value (wave 1 vs. wave 2) |
|  | No AKI (n=49) | | AKI  (n=132) | P value | No AKI (n=114) | | AKI (n=86) | P value |  |
| Difference in eGFR at baseline to 90 days  (mL/min/1.73m^2^) | 0.0  (-11.40, 15.69) | | 2.9  (-9.85, 16.28) | 0.438 | 4.87  (-1.56, 11.99) | | 3.34  (-7.96, 17.85) | 0.518 | 0.168 |
| Percentage of change in eGFR from baseline to 90 days (%) | 0.0  (12.50, 16.99) | | 3.79  (-13.19, 23.31) | 0.614 | 5.26  (-1.80, 14.92) | | 3.39  (-9.39, 23.79) | 0.541 | 0.135 |
| Difference in eGFR from discharge to 90 days  (mL/min/1.73m^2^) | -6.14  (-25.01, 0.00) | | 0.00  (-11.50, 5.10) | 0.0015 | -0.67  (-9.77, 1.82) | | 0.00  (-6.94, 6.36) | 0.192 | 0.217 |
| Percentage of change in eGFR from discharge to 90 days (%) | -5.15  (-21.67, 0.00) | | 0.00  (-11.74, 6.61) | 0.004 | -6.04  (-10.92, 1.84) | | 0.00  (-6.86, 7.26) | 0.218 | 0.232 |
| Excluded numbers: n=207 patients who had passed away, n=15 were dialysis dependent on discharge, n=7 were dialysis dependent at 90 days. N=543 had baseline and discharge eGFR values. N=381 have 90-day eGFR values.  For baseline eGFR n=48 values were imputed to 120 mL/min/1.73m^2^ (values above 120)  For discharge eGFR n=107 were imputed to 120 mL/min/1.73m^2^ (values above 120)  For 90-day eGFR n=51 were imputed to 120 mL/min/1.73m^2^ (values above 120) | | | | | | | | | |

Abbreviations: GFR, glomerular filtration rate; eGFR, estimated glomerular filtration rate; AKI, acute kidney injury

| **Table S9:** **Associations between AKI and KRT and 24-hour cumulative fluid balance** | | | | |
| --- | --- | --- | --- | --- |
|  | **AKI [odds ratio (95% CI)] (n=486)** | | **KRT [odds ratio (95% CI)] (n=664)** | |
|  | Unadjusted | Adjusted | Unadjusted | Adjusted |
| Fluid balance at 24 hours |  |  |  |  |
| < -2% (n=10, n=11) | 0.67 (0.18, 2.45) | 0.78 (0.19, 3.16) | Not enough data | Not enough data |
| -2% to 0% (n=220, n=275) | 0.72 (0.50, 1.05) | 0.83 (0.55, 1.26) | 0.59 (0.40, 0.88)* | 0.77 (0.48, 1.22) |
| 0% to +2% (n=227, n=323) | Reference | Reference | Reference | Reference |
| > +2% (n=29, n=66) | 1.65 (0.75, 3.65) | 1.36 (0.56, 3.30) | 2.19 (1.28, 3.74) ^*^ | 2.14 (1.16, 3.94)^*^ |
| Invasive ventilation | - | 1.10 (0.53, 2.28) | - | 2.94 (1.24, 6.96) ^*^ |
| Remdesivir | - | 0.85 (0.51, 1.40) | - | 0.56 (0.29, 1.10) |
| Steroids | - | 1.37 (0.73, 2.59) | - | 2.73 (1.48, 5.09) ^**^ |
| IL-6 antagonists | - | 0.65 (0.37, 1.14) | - | 0.76 (0.37, 1.55) |
| Non-renal SOFA score |  | 1.25 (1.11, 1.41)*** |  | 0.96 (0.85, 1.09) |
| Baseline diuretics |  | 1.12 (0.71, 1.76) |  | 0.82 (0.51, 1.30) |
| Max PEEP in first 24 hours |  | 1.06 (0.99, 1.12) |  | 1.00 (0.94, 1.06) |
| Vasopressor use |  | 0.66 (0.40, 1.06) |  | 1.11 (0.70, 1.76) |
| * <0.05 **<0.01 ***<0.001.  For the AKI model, there was available data for n=486, n=231 were excluded as they were diagnosed with AKI within 24 hours and a further 55 were excluded as they were missing cumulative fluid balance at 24hrs.  For the KRT model, there was available data for n=664, 26 were excluded as they were diagnosed with KRT within 24 hours and a further 82 were excluded as they were missing cumulative fluid balance at 24hrs. | | | | |

**Abbreviations:** SOFA, Sequential Organ Failure Assessment; CRP, c-reactive protein; AKI, acute kidney injury; KRT, kidney replacement therapy; PEEP, positive end-expiratory pressure; IL-6, interleukin 6
